# Gene Therapy and Cell-Based Therapies for Therapeutic Angiogenesis in Peripheral Artery Disease

**DOI:** 10.1155/2013/186215

**Published:** 2013-11-03

**Authors:** Munehisa Shimamura, Hironori Nakagami, Hiroshi Koriyama, Ryuichi Morishita

**Affiliations:** ^1^Division of Vascular Medicine and Epigenetics, Department of Child Development, United Graduate School of Child Development, Osaka University, Kanazawa University, and Hamamatsu University School of Medicine, 2-1 Yamadaoka, Suita 565-0817, Osaka, Japan; ^2^Department of Clinical Gene Therapy, Graduate School of Medicine, Osaka University, 2-2 Yamadaoka, Suita 565-0871, Osaka, Japan

## Abstract

Gene therapy and cell-based therapy have emerged as novel therapies to promote therapeutic angiogenesis in critical limb ischemia (CLI) caused by peripheral artery disease (PAD). Although researchers initially focused on gene therapy using proangiogenic factors, such as vascular endothelial growth factor (VEGF), fibroblast growth factor (FGF), and hepatocyte growth factors (HGF), cell therapy using bone marrow mononuclear cells (BMMNCs), mesenchymal stem cells (BMMSCs), G-CSF-mobilized peripheral blood mononuclear cells (M-PBMNCs), and endothelial progenitor cells (EPCs) have also been extensively studied. Based on the elaborate studies and favorable results of basic research, some clinical phase I/II trials have been performed, and the results demonstrate the safety of these approaches and their potential for symptomatic improvement in CLI. However, the phase 3 clinical trials have thus far been limited to gene therapy using the HGF gene. Further studies using well-designed larger placebo-controlled and long-term randomized control trials (RCTs) will clarify the effectiveness of gene therapy and cell-based therapy for the treatment of CLI. Furthermore, the development of efficient gene transfer systems and effective methods for keeping transplanted cells healthy will make these novel therapies more effective and ease the symptoms of CLI.

## 1. Introduction

Peripheral artery diseases (PAD), ischemic stroke, and coronary artery diseases refer to arterial stenosis caused by atherosclerosis and thrombosis. Critical limb ischemia (CLI) is a complication of PAD and causes pain on walking (claudication), pain at rest, and nonhealing ulcers. Although patients with CLI are treated with a combination of risk factor modification, such as statins, antiplatelet drugs, and angioplasty, these treatments are occasionally insufficient to recover sufficient blood flow to maintain normal tissue function. To overcome this limitation, therapeutic angiogenesis has emerged as a potential strategy to promote the growth of new vessels and thereby to supply sufficient blood flow. To date, researchers have focused on gene therapy using proangiogenic factors and/or cell-based therapy using several types of cells, including bone marrow cells (BMCs) and endothelial progenitor cells (EPCs), to achieve therapeutic angiogenesis. In gene therapy, the development of efficient gene transfer systems and investigation of suitable pro-angiogenic genes, such as vascular endothelial growth factor (VEGF), fibroblast growth factor (FGF), and hepatocyte growth factor (HGF), have been extensively studied in preclinical studies, whereas researchers in cell-based medicine have tried to find the most relevant cells and efficient methods for transplantation. Based on these results, clinical trials have been performed, and promising results have been reported. 

This review summarizes the basic aspects and clinical trials of therapeutic angiogenesis in PAD and discusses future directions.

## 2. Gene Therapy Using Proangiogenic Genes

Among pro-angiogenic genes, VEGF, a 45-kDa basic heparin that binds homodimeric glycoprotein, has been the most extensively studied. VEGF has 4 main isoforms: VEGF A, B, C, and D. There are additional isoforms in VEGF A: VEGF121, VEGF165, which is the most biologically active [1], VEGF189, and VEGF206. The receptors for VEGF are FLT-1 and FLK-1, which activate intracellular tyrosine kinase. Neuropilin 1 (NP-1) is another receptor for VEGF and is bound by VEGF165 [2]. NP-1 and FLK-1 are key mediators of the phosphoinositide-3-kinase and Akt (PI3K/Akt) and mitogen-activated protein kinase (MAPK) kinase pathways. The efficacy of therapeutic angiogenesis was initially reported using VEGF plasmid DNA gene transfer in human patients [3–5] (Table 1). An initial trial in 1994 used a hydrogel catheter with naked VEGF165 plasmid DNA and seemed to effectively stimulate collateral formation of blood vessels [3]. Intra-arterial administration into the site of percutaneous transluminal angioplasty (PTA) with adenoviruses or liposomes containing the VEGF165 gene was also reported to exhibit beneficial effects in increasing vascularity [6]. However, intramuscular injection of naked plasmids encoding the VEGF165 gene has also been attempted and reported to have beneficial effects in patients with peripheral arterial disease [4, 5] since many patients lack an appropriate target vascular lesion for catheter delivery. Adenovirus-mediated gene delivery of VEGF121 has also been reported to be effective in improving lower-extremity endothelial function and flow reserve [7]. Thus, gene therapy using the VEGF gene appears to be promising, but its efficacy remains controversial because two later randomized clinical trials (phase II) failed to meet the primary endpoint of significant amputation reduction [8] or a change in peak walking time (Delta PWT) at 12 weeks [9]. Although the former clinical trial exhibited benefits in the secondary endpoints of hemodynamic improvement, improvement in skin ulcers, and decreased pain [8], the latter clinical trial reported increased peripheral edema as well as no benefits in secondary endpoints such as DeltaPWT, the ankle-brachial index, claudication onset time, and quality-of-life measures [9]. Recently, Muona et al. reported a 10-year safety followup in patients that had undergone local VEGF gene transfer to ischemic lower limbs [10]. In the study, there were no differences in the causes of death or in the incidence of cancer or diabetic retinopathy between the control patients and the VEGF-treated patients. Furthermore, no significant differences were demonstrated in the number of amputations. From the viewpoint of the authors, treatment with VEGF gene transfer might not induce serious side effects but requires additional development to achieve further therapeutic effects.

FGF is another angiogenic factor that has been studied in PAD. There are at least 23 structurally related FGF proteins. Among them, FGF-1 (aFGF) and FGF-2 (bFGF) have been extensively studied. The safety and efficacy of increasing single and repeated doses of intramuscular naked plasmid DNA encoding FGF type 1 administered to patients with unreconstructable end-stage PAD was first shown in a phase I study [11]. In that study, a significant reduction in pain and aggregate ulcer size was detected after FGF gene transfer associated with an increased transcutaneous oxygen pressure (TcO2) and ankle pressure index (ABI) compared with baseline pretreatment values [11]. Furthermore, phase II trials demonstrated that NV1FGF-treated patients exhibited a significantly reduced risk of all amputations and major amputations and a trend towards a reduced risk of death, although improvements in ulcer healing were similar between the NV1FGF-treated group and the control group [12]. These results were promising, but recent large randomized phase III trials including 525 patients demonstrated no beneficial effects on either the secondary or primary endpoints, including reduction in time to amputation or death [14]. Thus, gene therapy using the FGF gene has been controversial thus far. 

Another promising pro-angiogenic factor is HGF, the efficacy of which has been reported in a phase III clinical trial [13]. HGF was first discovered as the most potent mitogen of hepatocytes, but it has been shown to possess multiple effects, including cell proliferation, angiogenesis, morphogenesis, anti-inflammation, and motility [16]. HGF exerts its angiogenic activity through tyrosine phosphorylation of its specific receptor, c-Met, which is expressed in endothelial cells (ECs) and vascular smooth muscle cells (VSMCs) [17]. Compared to bFGF, HGF can induce angiogenesis without the induction of vascular inflammation by nuclear factor-*κ*B (NF*κ*B)-induced interleukin-1 (IL-1) and monocyte chemotactic protein-1 (MCP-1) or vascular permeability through increased expression of aquaporin 1 (AQP1) [17]. Furthermore, gene transfer using naked plasmid HGF DNA was shown to induce therapeutic angiogenesis in animal models [18–23]. Based upon these findings, a human clinical trial (phase I/IIa) was started using intramuscular injection of naked human HGF plasmids [15]. Twenty-two patients with peripheral arterial disease or Buerger's disease staged as Fontaine IIb (*n* = 7), III (*n* = 4), or IV (*n* = 11) were treated with two injections of either 2 mg or 4 mg of HGF plasmid. No serious adverse events caused by gene transfer were detected over a followup of 6 months, and no peripheral edema was observed. Two months after gene transfer, the ankle-brachial index was increased. Additionally, the size of the largest ischemic ulcers and the visual analog scale score were decreased [15]. Recently, the long-term followup of this study was reported. An ankle-branchial pressure index >0.1 was observed in 11 of 14 patients (79%) at 2 years after gene therapy (11 of the 17 patients (65%) at 2 months). Reduction in rest pain (>2 cm in visual analog scale) was observed in 9 of 9 patients (100%) at 2 years (in 8 of 13 (62%) patients at 2 months). A reduction in ischemic ulcers accompanied by a decrease in the size of ulcers was observed in 9 of 10 patients (90%) at 2 years. Severe complications and adverse effects were not detected [24]. Powell et al. performed another double-blind placebo-controlled study with an HGF plasmid [25]. TcPO2 increased at 6 months in the high-dose group (4.0 mg at day 0, 14, 28) compared with the placebo, low-dose (0.4 mg at day 0, 14, 28), and middle-dose (4 mg at day 0, 28) groups, but there were no differences in the ankle-brachial index, toe-brachial index, pain relief, wound healing, or incidence of major amputation [25]. Finally, Shigematsu et al. performed a randomized, double-blind, placebo-controlled clinical trial of HGF plasmids in patients with PAD (phase III) [13]. The primary endpoint was improvement of rest pain in patients without ulcers (Rutherford 4) or a reduction of ulcer size in patients with ulcers (Rutherford 5). Secondary endpoints included ABI, amputation, and quality of life (QOL). Forty-four patients were recruited, and a significant difference in the primary endpoint was noted; improvement was observed in 70.4% of the HGF group and in 30.8% of the placebo group. When the analysis was limited to Rutherford 5 patients, HGF-treated patients exhibited a significantly higher improvement rate (100%) than did the placebo group (40%). QOL also improved in the HGF-treated group, and there were no major safety problems, although this trial failed to demonstrate an improvement of ABI or amputation rate. Following these favorable outcomes in HGF-treated patients, a global multicenter phase III clinical trial, which will recruit over 500 PAD patients, is scheduled.

Recently, one clinical trial (phase I) has reported the safety and possible efficacy of a plasmid HGF gene, VM202 [26], that encodes two isoforms of HGF, one consisting of 728 amino acids (known as HGF) and the other consisting of 723 amino acids (known as deleted HGF) [27]. In this trial, the median ABI and toe brachial pressure index (TBI) in the HGF-treated group were significantly increased at 12 months of followup, and the median visual analogue scale (VAS) decreased from 57.5 to 16.0 mm at 6 months of followup without significant differences in side effects. Based on these results, a phase 2 trial is ongoing (ClinicalTrials.gov NCT01064440).

Thus, the effectiveness of gene therapy using pro-angiogenic factors remains controversial, but some clinical trials have shown promising results. Further large-scale clinical trials will clarify their efficacy.

## 3. Cell-Based Therapy in PAD

While the development of gene therapy has been ongoing, researchers have attempted to develop more effective treatments (Table 2). On such potential treatment is the transplantation of stem or progenitor cells, which possess the capability to self-renew and to differentiate into organ-specific cell types as well as to mediate paracrine effects through the release of pro-angiogenic growth factors. The cells that have been used in these studies include bone marrow mononuclear cells (BMMNCs), bone marrow mesenchymal stem cells (BMMSCs), G-CSF-mobilized peripheral blood mononuclear cells (M-PBMNCs), endothelial progenitor cells (EPCs), and G-CSF monotherapy.

Broadly, bone marrow cells (BMCs) are harvested from bone marrow and can be identified as crude, unfractionated, or mononuclear cells (BMMNCs) by density centrifugation [42]. Although BMCs contain MSCs and EPCs as well as hematopoietic stem cells and hemangioblasts, EPCs can also be isolated from the peripheral blood. G-CSF can mobilize peripheral blood CD34^+^, CD133^+^, and/or KDR^+^ cells with the capacity to differentiate into EPCs [30].

### 3.1. Clinical Trials of Therapy Using Bone Marrow Mononuclear Cells, Mesenchymal Stem Cells, or Peripheral Blood Mononuclear Cells

The Angiogenesis using cell transplantation (TACT) study demonstrated that intramuscular injection of the mononuclear fraction of autologous BMCs into affected areas in patients with unilateral ischemia of the leg (25 patients) and one leg in bilateral leg ischemia (22 patients) increased perfusion with significant improvements in the ankle-brachial indices, TcO2, and rest pain in injected legs at 4 weeks and that recovery was sustained for 24 weeks [28]. Following this study, some phase I clinical trials have explored the efficacy of BMMNCs in improving limb salvage in patients with CLI [29, 31, 32]. Thus, initial studies have extensively examined the effectiveness of transplantation of BMCs and the best methodology, such as the source of cells and delivery route. 

Regarding the association between the subpopulations of BMMNCs and outcomes, a phase I open-label, nonrandomized trial reported a significant difference in counts of KDR^+^ cells between treatment responders and nonresponders, although no correlation was observed between total mononuclear cell count and changes in ABI [37]. Additionally, improvements in limb perfusion were associated with KDR^+^ but not CD34^+^ or CD133^+^ subpopulations of BMMNCs [37]. In this trial, the amputation-free survival at 1 year was 86.3% with significant recovery in first toe pressure, TBI, perfusion index, rest pain, and QOL and a trend towards improvement in ABI [37]. Another study using BMMNCs reported that bone marrow lymphopenia in the initial bone marrow concentrates in patients might be potential causative factors for failure of BMMNC therapy, suggesting that at least partial correction with platelet supplementation may be beneficial [36]. In this trial, a total of 96 patients were randomized into a BMMNC treatment group or a standard medical care group. The frequency of major limb amputation was 21% in the BMMNC group and 44% in the control group within the 120 days of followup [36].

To identify better cells for the treatment of diabetic CLI and foot ulcers in a pilot trial, a double-blind, randomized, controlled trial compared the effectiveness of BMMSCs and BMMNCs [38]. The ulcer healing rate of the BMMSC group was significantly higher than that of the BMMNC group at 6 weeks after injection and reached 100% four weeks earlier than the BMMNC group. After 24 weeks of followup, the improvements in limb perfusion induced by the BMMSC transplantation were more significant than those induced by BMMNC transplantation in terms of painless walking time, ABI, TcO2, and magnetic resonance angiography (MRA) analysis [38]. The authors concluded that BMMSC therapy might be better tolerated and more effective than BMMNC therapy for increasing lower limb perfusion and promoting foot ulcer healing in diabetic patients with CLI [38]. Another randomized trial, which recruited 150 patients, was performed to compare the effectiveness of M-PBMNCs and BMMNCs [34]. Seventy-six patients received M-PBMNCs, and 74 patients received BMMNCs; the groups were followed up for 12 weeks. Twelve weeks after cell implantation, ABI, skin temperature, and rest pain were significantly better in M-PBMNC-treated patients than in BMMNC-treated patients. However, no significant differences were observed in pain-free walking distance, TcO2, ulcers, or the rate of lower limb amputation between the two groups [34]. The authors concluded that M-PBMNC treatment would be more practical than treatment with BMMNCs [34].

Regarding the administration route, cells were injected i.m. in most studies, but the efficacy of intra-arterial injection was also examined in the intra-arterial progenitor cell transplantation of bone marrow mononuclear cells for induction of neovascularization in patients with peripheral arterial occlusive disease (PROVASA) study [39]. In this multicenter, phase II, double-blind, randomized-start trial, forty patients with CLI were included and received intra-arterial administration of either BM-MNC or placebo followed by active treatment with BM-MNC (open label) after 3 months. As a result, intra-arterial administration of BMMNCs did not achieve the primary endpoint, which was an increase in ABI. However, cell therapy resulted in improved ulcer healing versus placebo within 3 months, although limb salvage and amputation-free survival rates did not differ between the groups. Repeated BMMNC administration correlated significantly with limb salvage [39]. 

To minimize the invasiveness of BM absorption, cellular therapy with Ixmyelocel-T and treatment with commercial preexpanded cells obtained from a small amount of a subject's own bone marrow under conscious sedation were evaluated in a prospective, randomized, double-blind, placebo-controlled, multicenter study (RESTORE-CLI) [40]. Patients with lower extremity CLI with no options for revascularization received single injections into one leg and were followed for 12 months. Ixmyelocel-T treatment resulted in a significantly prolonged time to the first occurrence of treatment failure (major amputation of injected leg, all-cause mortality, doubling of the total wound surface area from baseline, or de novo gangrene). There was a trend towards increased amputation-free survival after Ixmyelocel-T treatment, but the trend was not statistically significant. The treatment effect in the post hoc analyses of patients with baseline wounds was more pronounced [40].

G-CSF monotherapy is one treatment that can avoid the invasiveness of bone marrow transplantation [33]. Thirty-nine patients were randomly assigned to conventional drug therapy, conventional drug therapy plus bone marrow transplantation (BMT), or conventional therapy plus G-CSF. Subjective symptoms, ABI, and TcO2 were significantly improved in the G-CSF and BMT groups to the same degree, whereas such improvements were not observed in the conventional therapy group [33].

### 3.2. Meta-Analysis in BMC- and M-PBMNC-Based Clinical Trials

Based on these clinical trials, meta-analyses or systematic reviews have been reported recently [43–45]. One meta-analysis of 37 controlled and noncontrolled, randomized and nonrandomized trials demonstrated that autologous BMCs or M-PBMNCs were effective in improving surrogate indexes of ischemia, subjective symptoms, and ulcer healing and amputations, whereas G-CSF monotherapy did not result in significant improvements in the same endpoints [43]. Furthermore, this study demonstrated that cell-based treatment was more effective in patients with Buerger's disease than in those with atherosclerotic PAD. The intramuscular route was better than the intra-arterial route, and the use of BMCs was better than the use of M-PBMNCs. Another recent systematic analysis of 45 cell-based clinical trials and seven non-placebo-controlled and placebo-controlled RCTs reported that cell therapy using BMMNCs or M-PBMNCs resulted in a favorable safety profile with a low adverse event rate, no increase in severe events such as mortality and cancer, and that cell therapy decreased the risk of amputation [44]. No difference in the amputation rate between BMMNC therapy and M-PBMNC therapy was observed [44]. Thus, these meta-analyses demonstrated the feasibility of cell-based therapy, but there are some discrepancies in their findings and those of individual clinical trials regarding the best source of cells and the best route of delivery. 

Most recently, a meta-analysis of 12 randomized controlled clinical trials was reported [45]. This meta-analysis studied BM-derived cell therapy compared with standard care with or without placebo in 510 CLI subjects, including 7 trials using BMMNC, 3 trials using BMMSC, 2 trials using M-PBMNC, and 1 trial using Ixmyelocel-T. When major amputation and amputation-free survival were considered as the primary endpoints, beneficial effects of BM-derived cell therapy were observed for both subjective and surrogate objective endpoints, including pain score, pain-free walking distance, ankle-brachial index, and TcO2 measurements. However, when the analysis was limited to the 7 placebo-controlled RCTs, the beneficial effect on major amputation rates and amputation-free survival was reduced and not significant, indicating that a placebo in the control arms is necessary. This result indicates that well-designed larger placebo-controlled RCTs including long-term followup data are needed to confirm the effects of BMC and M-PBMNC treatments [45].

### 3.3. EPCs

EPCs were first described by Asahara et al. as circulating CD34^+^ cells that could differentiate into endothelial cells (ECs) and incorporate into foci of neovascularization [46]. Recent studies have described 4 sources of EPCs: hematopoietic stem cells; myeloid cells; other circulating cells, termed “side population cells”; and circulating mature endothelial cells that have sheared off from vessel walls [47]. Although Asahara et al. first used CD34 and VEGF receptor-2 to discriminate EPCs [46], subsequent studies have shown that specific cell markers or functions of EPCs remain controversial [47] because hematopoietic stem cells also express CD34, CD133, and VEGF receptor-2 [48]. However, recent studies have used CD34^+^ or CD133^+^ cells in preclinical and clinical studies in PAD and have reported their effectiveness.

One such clinical study is a phase I/IIa, multicenter, single-blinded, dose-escalation clinical trial of autologous CD34^+^ cells, which include the endothelial and hematopoietic progenitor-enriched fraction, in no-option patients with atherosclerotic peripheral artery disease or Buerger's disease with CLI [35]. CD34^+^ cells isolated from the G-CSF-mobilized apheresis product were injected i.m. into the leg with more severe ischemia. CD34^+^ cell-treated patients demonstrated significant recovery in the primary endpoints, including the efficacy score, representing changes in the toe brachial pressure index (TBPI), the Wong-Baker FACES pain rating scale, and the total walking distance 12 weeks after cell transplantation without a significant dose-response relationship. During the 12-week followup, no death or major amputation occurred [35]. Recently, the long-term outcome for these patients was analyzed. The incidence of major clinical events and physiological parameters of limb ischemia were evaluated up to 208 weeks after the therapy [49]. Three patients with PAD died by week 156, and 1 patient with Buerger's disease died by week 208, due to cardiac complications. No patients underwent major amputation, although 1 patient with Buerger's disease underwent minor amputation by week 104. The toe brachial pressure index versus the baseline was sustained up to week 208 and that of transcutaneous partial oxygen pressure was maintained up to week 156. Measures of functional recovery, such as the Wong-Baker FACES pain rating scale, ulcer size, and exercise tolerance, were significantly improved compared with baseline [49].

Most recently, a randomized, double-blind, placebo-controlled pilot study was performed to examine the safety and efficacy of intramuscular injections of autologous CD34^+^ cells in 28 patients with moderate- or high-risk CLI [41]. There was a trend towards a reduction in amputation rates at 6 and 12 months after treatment without adverse safety signals associated with cell administration, although several surrogate markers, such as ABI, toe brachial index, leg pain, walking distance, and wound healing, did not exhibit differences because of low statistical power [41]. No adverse events associated with cell transplantation were observed [41]. 

The effect of autologous peripheral blood CD133^+^ cell implantation was also reported in small cohort study, including 7 patients suffering from ASO, one with Buerger's disease, and one with thromboembolic disorder [50]. CD133^+^ cells, which were selected from autologous PBSCs collected after the administration of G-CSF, were administered i.m. After 1 year, seven of nine patients were free from leg amputation, and there was a trend towards improvement in pain-free treadmill walking time and exercise capacity [50]. 

Thus, EPC-based treatment seems also beneficial, but further large-scale studies will clarify the efficacy of EPC transplantation.

## 4. Conclusion

Overall, clinical trials have demonstrated that gene therapy and cell-based therapy may be safe and effective in the treatment of CLI, although gene therapy using HGF remains a phase III-proven therapy, where the number of recruited patients is not large. A recent meta-analysis in BMCs and M-PBMNCs demonstrated that the beneficial effect on major amputation rates and amputation-free survival was reduced and nonsignificant in the placebo-controlled RCTs [45]; well-designed larger placebo-controlled RCTs are required to establish the efficacies of these novel therapies. Furthermore, the long-term effects of these therapies should be verified. 

Also, additional improvement should be pursued to achieve more efficient therapy. Recently, the proliferative and migratory function in EPCs in diabetic patients has been report to be reduced [51]. One of the mechanisms in EPCs dysfunction is associated with defective NO signaling [52]. To overcome the dysfunction, inhibition of NADPH oxidase was reported to restore NO availability and migratory function in diabetic CD34 cells [53]. Thus, adjuvant therapy to promote BMC and EPC health is one of solutions to make the cell-based therapy more effective. In the field of gene therapy, improvements in efficient gene transfer systems are required. These include improvement of the development of devices as well as the structure of vectors. For example, ultrasound-microbubbles-mediated gene transfer is one of such devices that could increase the transfection efficiency of naked plasmid DNA and is shown to enhance angiogenesis in ischemic limb in rodent [54]. 

We believe that these basic, translational, and clinical studies will lead to improvements in QOL for PAD patients.

## Figures and Tables

**Table 1:** Clinical trials of gene therapy in peripheral artery diseases.

Authors	Year	Gene	Vector	Delivery route	*n*	Reference number
Baumgartner et al.	1998	VEGF_165_	naked pDNA	IM	9	[4]
Isner et al.	1998	VEGF_165_	naked pDNA	IM	6	[5]
Rajagopalan et al.	2001	VEGF_121_	adenovirus	IM	5	[7]
Mäkinen et al.	2002	VEGF_165_	adenovirus, plasmid/liposome	IA	54	[6]
Comerota et al.	2002	FGF-1	naked pDNA	IA	51	[11]
Rajagopalan et al.	2003	VEGF_121_	adenovirus	IM	1 : 1 : 1 fashion to low dose, high dose, or placebo arms (35-36 patients in each group)	[9]
Kusumanto et al.	2006	VEGF_165_	naked pDNA	IM	54	[8]
Nikol et al.	2008	FGF-1	naked pDNA	IM	125	[12]
Shigematsu et al.	2010	HGF	naked pDNA	IM	44	[13]
Belch et al.	2011	FGF-1	naked pDNA	IM	525	[14]
Morishita et al.	2011	HGF	naked pDNA	IM	22	[15]

VEGF: vascular endothelial growth factor; FGF: fibroblast growth factor (FGF); HGF: hepatocyte growth factors.

**Table 2:** Clinical trials of cell-based therapy in peripheral artery diseases.

Authors	Year	Cell	Delivery route	*n*	Reference number
Tateishi-Yuyama et al.	2002	BMMNC or PBMNC	IM	45	[28]
Esato et al.	2002	BMC	IM	8	[29]
Huang et al.	2005	PBMNC	IM	28	[30]
Miyamoto et al.	2006	BMMNC	IM	8	[31]
Durdu et al.	2006	BMMNC	IM	28	[32]
Arai et al.	2006	G-CSF	SC	39	[33]
Huang et al.	2007	BMMNC or PBMNC	IM	150	[34]
Kawamoto et al.	2009	EPC	IM	17	[35]
Procházka et al.	2010	BMC	IM	96	[36]
Murphy et al.	2011	BMMNC	IM	29	[37]
Lu et al.	2011	BMMNC or BMMSC	IM	41	[38]
Walter et al.	2011	BMMNC	IA	40	[39]
Powell et al.	2012	Ixmyelocel-T	IM	72	[40]
Losordo et al.	2012	EPC	IM	28	[41]

PBMNC: peripheral blood mononuclear cells; BMC: bone marrow cell; BMMNC: bone marrow-derived mononuclear cell; BMMSC: bone marrow mesenchymal stem cell; G-CSF: granulocyte colony-stimulating factor; EPC: endothelial progenitor cell.

## References

[1] Ferrara N, Gerber H-P, LeCouter J (2003). The biology of VEGF and its receptors. *Nature Medicine*.

[2] Ferrara N, Alitalo K (1999). Clinical applications of angiogenic growth factors and their inhibitors. *Nature Medicine*.

[3] Isner JM, Pieczek A, Schainfeld R (1996). Clinical evidence of angiogenesis after arterial gene transfer of phVEGF165 in patient with ischaemic limb. *The Lancet*.

[4] Baumgartner I, Pieczek A, Manor O (1998). Constitutive expression of phVEGF165 after intramuscular gene transfer promotes collateral vessel development in patients with critical limb ischemia. *Circulation*.

[5] Isner JM, Baumgartner I, Rauh G (1998). Treatment of thromboangiitis obliterans (Buerger’s disease) by intramuscular gene transfer of vascular endothelial growth factor: preliminary clinical results. *Journal of Vascular Surgery*.

[6] Mäkinen K, Mannine H, Hedman M (2002). Increased vascularity detected by digital subtraction angiography after VEGF gene transfer to human lower limb artery: a randomized, placebo-controlled, double-blinded phase II study. *Molecular Therapy*.

[7] Rajagopalan S, Shah M, Luciano A, Crystal R, Nabel EG (2001). Adenovirus-mediated gene transfer of VEGF121 improves lower-extremity endothelial function and flow reserve. *Circulation*.

[8] Kusumanto YH, van Weel V, Mulder NH (2006). Treatment with intramuscular vascular endothelial growth factor gene compared with placebo for patients with diabetes mellitus and critical limb ischemia: a double-blind randomized trial. *Human Gene Therapy*.

[9] Rajagopalan S, Mohler ER, Lederman RJ (2003). Regional angiogenesis with vascular endothelial growth factor in peripheral arterial disease: a phase II randomized, double-blind, controlled study of adenoviral delivery of vascular endothelial growth factor 121 in patients with disabling intermittent claudication. *Circulation*.

[10] Muona K, Mäkinen K, Hedman M, Manninen H, Ylä-Herttuala S (2012). 10-Year safety follow-up in patients with local VEGF gene transfer to ischemic lower limb. *Gene Therapy*.

[11] Comerota AJ, Throm RC, Miller KA (2002). Naked plasmid DNA encoding fibroblast growth factor type 1 for the treatment of end-stage unreconstructible lower extremity ischemia: preliminary results of a phase I trial. *Journal of Vascular Surgery*.

[12] Nikol S, Baumgartner I, van Belle E (2008). Therapeutic angiogenesis with intramuscular NV1FGF improves amputation-free survival in patients with critical limb ischemia. *Molecular Therapy*.

[13] Shigematsu H, Yasuda K, Iwai T (2010). Randomized, double-blind, placebo-controlled clinical trial of hepatocyte growth factor plasmid for critical limb ischemia. *Gene Therapy*.

[14] Belch J, Hiatt WR, Baumgartner I (2011). Effect of fibroblast growth factor NV1FGF on amputation and death: a randomised placebo-controlled trial of gene therapy in critical limb ischaemia. *The Lancet*.

[15] Morishita R, Makino H, Aoki M (2011). Phase I/IIa clinical trial of therapeutic angiogenesis using hepatocyte growth factor gene transfer to treat critical limb ischemia. *Arteriosclerosis, Thrombosis, and Vascular Biology*.

[16] Nakamura T, Mizuno S (2010). The discovery of Hepatocyte Growth Factor (HGF) and its significance for cell biology, life sciences and clinical medicine. *Proceedings of the Japan Academy B*.

[17] Kaga T, Kawano H, Sakaguchi M, Nakazawa T, Taniyama Y, Morishita R (2012). Hepatocyte growth factor stimulated angiogenesis without inflammation: differential actions between hepatocyte growth factor, vascular endothelial growth factor and basic fibroblast growth factor. *Vascular Pharmacology*.

[18] Morishita R, Nakamura S, Hayashi S-I (1999). Therapeutic angiogenesis induced by human recombinant hepatocyte growth factor in rabbit hind limb ischemia model as cytokine supplement therapy. *Hypertension*.

[19] Hayashi S-I, Morishita R, Nakamura S (1999). Potential role of hepatocyte growth factor, a novel angiogenic growth factor, in peripheral arterial disease: downregulation of HGF in response to hypoxia in vascular cells. *Circulation*.

[20] Taniyama Y, Morishita R, Hiraoka K (2001). Therapeutic angiogenesis induced by human hepatocyte growth factor gene in rat diabetic hind limb ischemia model: molecular mechanisms of delayed angiogenesis in diabetes. *Circulation*.

[21] Taniyama Y, Morishita R, Aoki M (2001). Therapeutic angiogenesis induced by human hepatocyte growth factor gene in rat and rabbit hindlimb ischemia models: preclinical study for treatment of peripheral arterial disease. *Gene Therapy*.

[22] Hiraoka K, Koike H, Yamamoto S (2003). Enhanced therapeutic angiogenesis by cotransfection of prostacyclin synthase gene or optimization of intramuscular injection of naked plasmid DNA. *Circulation*.

[23] Koike H, Morishita R, Iguchi S (2003). Enhanced angiogenesis and improvement of neuropathy by cotransfection of human hepatocyte growth factor and prostacyclin synthase gene. *The FASEB Journal*.

[24] Makino H, Aoki M, Hashiya N (2012). Long-term follow-up evaluation of results from clinical trial using hepatocyte growth factor gene to treat severe peripheral arterial disease. *Arteriosclerosis, Thrombosis, and Vascular Biology*.

[25] Powell RJ, Simons M, Mendelsohn FO (2008). Results of a double-blind, placebo-controlled study to assess the safety of intramuscular injection of hepatocyte growth factor plasmid to improve limb perfusion in patients with critical limb ischemia. *Circulation*.

[26] Henry TD, Hirsch AT, Goldman J (2011). Safety of a non-viral plasmid-encoding dual isoforms of hepatocyte growth factor in critical limb ischemia patients: a phase I study. *Gene Therapy*.

[27] Saeed M, Martin A, Ursell P (2008). MR assessment of myocardial perfusion, viability, and function after intramyocardial transfer of VM202, a new plasmid human hepatocyte growth factor in ischemic swine myocardium. *Radiology*.

[28] Tateishi-Yuyama E, Matsubara H, Murohara T (2002). Therapeutic angiogenesis for patients with limb ischaemia by autologous transplantation of bone-marrow cells: a pilot study and a randomised controlled trial. *The Lancet*.

[29] Esato K, Hamano K, Li T-S (2002). Neovascularization induced by autologous bone marrow cell implantation in peripheral arterial disease. *Cell Transplantation*.

[30] Huang P, Li S, Han M, Xiao Z, Yang R, Han ZC (2005). Autologous transplantation of granulocyte colony-stimulating factor-mobilized peripheral blood mononuclear cells improves critical limb ischemia in diabetes. *Diabetes Care*.

[31] Miyamoto K, Nishigami K, Nagaya N (2006). Unblinded pilot study of autologous transplantation of bone marrow mononuclear cells in patients with thromboangiitis obliterans. *Circulation*.

[32] Durdu S, Akar AR, Arat M, Sancak T, Eren NT, Ozyurda U (2006). Autologous bone-marrow mononuclear cell implantation for patients with Rutherford grade II-III thromboangiitis obliterans. *Journal of Vascular Surgery*.

[33] Arai M, Misao Y, Nagai H (2006). Granulocyte colony-stimulating factor—a noninvasive regeneration therapy for treating atherosclerotic peripheral artery disease. *Circulation Journal*.

[34] Huang PP, Yang XF, Li SZ, Wen JC, Zhang Y, Han ZC (2007). Randomised comparison of G-CSF-mobilized peripheral blood mononuclear cells versus bone marrow-mononuclear cells for the treatment of patients with lower limb arteriosclerosis obliterans. *Thrombosis and Haemostasis*.

[35] Kawamoto A, Katayama M, Handa N (2009). Intramuscular transplantation of G-CSF-mobilized CD^34+^ cells in patients with critical limb ischemia: a phase I/IIa, multicenter, single-blinded, dose-escalation clinical trial. *Stem Cells*.

[36] Procházka V, Gumulec J, Jalůvka F (2010). Cell therapy, a new standard in management of chronic critical limb ischemia and foot ulcer. *Cell Transplantation*.

[37] Murphy MP, Lawson JH, Rapp BM (2011). Autologous bone marrow mononuclear cell therapy is safe and promotes amputation-free survival in patients with critical limb ischemia. *Journal of Vascular Surgery*.

[38] Lu D, Chen B, Liang Z (2011). Comparison of bone marrow mesenchymal stem cells with bone marrow-derived mononuclear cells for treatment of diabetic critical limb ischemia and foot ulcer: a double-blind, randomized, controlled trial. *Diabetes Research and Clinical Practice*.

[39] Walter DH, Krankenberg H, Balzer JO (2011). Intraarterial administration of bone marrow mononuclear cells in patients with critical limb ischemia a randomized-start, placebo-controlled pilot trial (PROVASA). *Circulation*.

[40] Powell RJ, Marston WA, Berceli SA (2012). Cellular therapy with Ixmyelocel-T to treat critical limb ischemia: the randomized, double-blind, placebo-controlled RESTORE-CLI trial. *Molecular Therapy*.

[41] Losordo DW, Kibbe MR, Mendelsohn F (2012). A randomized, controlled pilot study of autologous CD^34+^ cell therapy for critical limb ischemia. *Circulation*.

[42] Ouma GO, Jonas RA, Usman MH, Mohler ER (2012). Targets and delivery methods for therapeutic angiogenesis in peripheral artery disease. *Vascular Medicine*.

[43] Fadini GP, Agostini C, Avogaro A (2010). Autologous stem cell therapy for peripheral arterial disease. Meta-analysis and systematic review of the literature. *Atherosclerosis*.

[44] Benoit E, O’Donnell TF, Patel AN (2013). Safety and efficacy of autologous cell therapy in critical limb ischemia: a systematic review. *Cell Transplantation*.

[45] Teraa M, Sprengers RW, van der Graaf Y, Peters CE, Moll FL, Verhaar MC (2013). Autologous bone marrow-derived cell therapy in patients with critical limb ischemia: a meta-analysis of randomized controlled clinical trials. *Annals of Surgery*.

[46] Asahara T, Murohara T, Sullivan A (1997). Isolation of putative progenitor endothelial cells for angiogenesis. *Science*.

[47] Matthias M, David N, Josef N (2011). From bench to bedside: what physicians need to know about endothelial progenitor cells. *American Journal of Medicine*.

[48] Raval Z, Losordo DW (2013). Cell therapy of peripheral arterial disease from experimental findings to clinical trials. *Circulation Research*.

[49] Kinoshita M, Fujita Y, Katayama M (2012). Long-term clinical outcome after intramuscular transplantation of granulocyte colony stimulating factor-mobilized CD^34^ positive cells in patients with critical limb ischemia. *Atherosclerosis*.

[50] Burt RK, Testori A, Oyama Y (2010). Autologous peripheral blood CD^133+^ cell implantation for limb salvage in patients with critical limb ischemia. *Bone Marrow Transplantation*.

[51] Tepper OM, Galiano RD, Capla JM (2002). Human endothelial progenitor cells from type II diabetics exhibit impaired proliferation, adhesion, and incorporation into vascular structures. *Circulation*.

[52] Jarajapu YPR, Grant MB (2010). The promise of cell-based therapies for diabetic complications: challenges and solutions. *Circulation Research*.

[53] Jarajapu YPR, Caballero S, Verma A (2011). Blockade of NADPH oxidase restores vasoreparative function in diabetic CD^34+^ cells. *Investigative Ophthalmology & Visual Science*.

[54] Taniyama Y, Tachibana K, Hiraoka K (2002). Development of safe and efficient novel nonviral gene transfer using ultrasound: enhancement of transfection efficiency of naked plasmid DNA in skeletal muscle. *Gene Therapy*.

